# Millennia-old coral holobiont DNA provides insight into future adaptive trajectories

**DOI:** 10.1111/mec.16642

**Published:** 2022-08-18

**Authors:** Carly B. Scott, Anny Cárdenas, Matthew Mah, Vagheesh M. Narasimhan, Nadin Rohland, Lauren T. Toth, Christian R. Voolstra, David Reich, Mikhail V. Matz

**Affiliations:** 1 Department of Integrative Biology, University of Texas, Austin, TX, USA; 2 Department of Biology, University of Konstanz, Konstanz, Germany; 3 Department of Genetics, Harvard Medical School, Boston, MA, USA; 4 Broad Institute of Harvard and MIT, Cambridge, MA, USA; 5 Howard Hughes Medical Institute, Harvard Medical School, Boston, MA, USA; 6 Department of Statistics and Data Science, University of Texas, Austin, TX, USA; 7 U.S. Geological Survey, St. Petersburg Coastal and Marine Science Center, St. Petersburg, FL; 8 Department of Human Evolutionary Biology, Harvard University, Cambridge, MA, USA; 9 Lead contact

**Keywords:** Ancient DNA, coral, genomics, holobiont evolution, microbial diversity, reef paleoecology

## Abstract

Ancient DNA (aDNA) has been applied to evolutionary questions across a wide variety of taxa. Here, for the first time, we leverage aDNA from millennia-old fossil coral fragments to gain new insights into a rapidly declining western Atlantic reef ecosystem. We sampled four *Acropora palmata* fragments (dated 4215 BCE - 1099 CE) obtained from two Florida Keys reef cores. From these samples, we established that it is possible both to sequence ancient DNA from reef cores and place the data in the context of modern-day genetic variation. We recovered varying amounts of nuclear DNA exhibiting the characteristic signatures of aDNA from the *A. palmata* fragments. To describe the holobiont *sensu lato*, which plays a crucial role in reef health, we utilized metagenome-assembled genomes as a reference to identify a large additional proportion of ancient microbial DNA from the samples. The samples shared many common microbes with modern-day coral holobionts from the same region, suggesting remarkable holobiont stability over time. In contrast, we were unable to recovery any ancient *Symbiodiniaceae* reads. Comparing the ancient *A. palmata* data to whole-genome sequencing data from living acroporids, we found that while slightly distinct, ancient samples were most closely related to individuals of their own species. Together, these results provide a proof-of-principle showing that it is possible to carry out direct analysis of coral holobiont change over time, which lays a foundation for studying the impacts of environmental stress and evolutionary constraints.

## Introduction

Applications of ancient DNA (aDNA) to ecological and evolutionary questions have been conducted across a variety of taxa^1,2^ - from horses^3^ to humans^4^. One of the most successful marine applications of aDNA has been to the carbonate shells of marine molluscs, where nuclear DNA over 100,000 years old was recovered and compared to extant molluscs^1^. Here, we apply this technology to scleractinian corals whose populations are declining on a global scale as a result of climate change and other anthropogenic disturbances^5,6^. Although the coral animals themselves die and degrade over time, the calcium carbonate skeleton corals produce remains on the reef, providing a fossil record of long-term reef evolution. This creates the potential for skeletons of long-dead corals extracted from reef cores to preserve DNA, suitable and sufficient for aDNA analysis.

Beyond the coral themselves, the microbes and dinoflagellate Symbiodiniaceae associated with corals constitute a dynamic system which forms the coral holobiont or metaorganism that determines coral resilience^7^. Several projects^8–11^ have aimed to capture microbial diversity through the construction of metagenome-assembled genomes (MAGs)^12–14^. MAGs are assembled from shotgun sequence data from environmental or host-centric samples, in contrast to genomes assembled from lab cultures, reducing bias towards highly studied (or cultivable) microbes^10^. Further, MAGs have successfully been constructed from ancient material^15^, suggesting we may be able to recover coral-associated microbes from the past.

There have been several studies that have examined the population genetics of coral from multiple modern-day reefs including from the Florida Keys as well as from the Great Barrier Reef. These large-scale sampling experiments involved dozens of individuals collected from multiple reef sites and have documented population structure^16–18^, hybridization^19^, and elucidated the genomic basis of thermal tolerance^18,20^. These reef- and basin-wide genetic studies have informed predictions of which extant coral populations are most vulnerable to climate change^21^. While present-day genetic diversity and distribution has been assessed in coral, no aDNA based studies have yet been carried out to examine long-term demographic trends or to understand how corals have responded broadly to global change that has occurred over the past few thousand years.

In this study we were interested in three related scientific goals. First, we aimed to extend the genetic analysis of coral to ancient time periods by establishing that we could successfully recover nuclear DNA from corals in reef cores that were thousands of years old. Second, we wanted to compare the data from ancient corals to those from extant populations to place both coral and microbial aDNA in the context of present-day genetic variation. Third, we wanted to utilize MAGs in order to assess microbial species richness within ancient coral holobionts and to examine if there had been changes in the distribution of these holobionts.

## Materials & Methods

### Sampling

The ancient coral samples were obtained from reef cores in the U.S. Geological Survey (USGS) Core Archive housed at the USGS Coastal and Marine Science Center in St. Petersburg, Florida. Cores were initially collected using the USGS wireline hydraulic drilling system^51^. The cores from Looe Key were collected in 1981 and the core from Sombrero Reef was collected in 2015^51,52^. The ages of the corals were determined by radiocarbon dating^51,52^. Samples for aDNA analysis were cut from the internal skeletons of the corals using a tile saw dedicated to that purpose. Those samples were then individually sonicated in warm reverse osmosis water to remove any macroscopic surface contamination, dried overnight, and sealed in sterile sample bags prior to analysis. Information on all samples is listed in Table S1.

Samples were sent to Harvard Medical School, where sample processing, DNA extraction and library preparation happened in dedicated aDNA clean rooms. Samples were subjected to ultraviolet light (254nm for 2 × 5min) for surface decontamination and subsequently milled in a Retsch Mixer Mill.

### DNA Extraction

DNA was extracted for high throughput sequencing according to Rohland *et al*.^53^. All extractions were performed in a clean room, and no other coral processing had been done there prior. This protocol is optimized for the retrieval of short DNA fragments using a silica-based extraction method. Our 4 samples were processed in this fashion using DNA binding buffer D (5 M guanidine hydrochloride, 40% vol/vol 2-propanol, 0.12 M sodium acetate and 0.05% vol/vol Tween 20) and silica beads.

### Library Preparation & Sequencing

Libraries were prepared for sequencing as in Rohland et al.^26^ We applied partial uracil-DNA-glycosylase treatment to libraries to leverage the advantages of retaining some characteristic aDNA patterns for downstream authentication while still repairing most damage in ancient molecules to reduce biases during analysis. Samples were sequenced in 2019 using an Illumina HiSeq X 10 instrument to obtain 2 × 101 bp paired end reads and 2 × 7 bp for the index reads.

### Read processing

Paired-end reads were merged to single-end reads before alignment to filter out long fragments and trim adapters. A minimum of 15 base-pairs of overlap was required, allowing for up to one mismatch at positions with base quality of at least 20, and up to three mismatches at positions of lower base quality. At mismatch positions, the base call of higher quality was retained, and the corresponding base quality was the difference of the two base qualities. For matching positions, the base quality was the maximum of the two base qualities.

Modern *Acropora* spp. samples (N = 60) were retrieved for comparison from bioproject PRJNA473816^16^. These samples span five locations: Belize, the Bahamas, Curacao, the US Virgin Islands, and the Florida Keys. Modern-day microbial samples (N = 81) from two Florida Keys reefs (Looe Key and Molasses Reef) were retrieved from bioproject PRJNA299413^31^. An additional 96 microbial samples from Florida Keys acroporids were retrieved from bioproject PRJNA546259^32^. Modern comparison samples are summarized in Table S3. All modern samples were aligned to the master reference, described below, using “local” bowtie2 mapping parameters.

The sequences from all samples were mapped to a concatenated reference genome of *A. millepora*^20^*, Symbiodinium* sp.^54^*, Breviolum minutum*^55^*, Cladocopium* sp.^54^*, Durusdinium* sp. (Unpublished; Dougan, Bellantuono, Granados-Cifuentes, and Rodriguez-Lanetty), and 103 assorted bacterial genomes (Associated with this paper; Voolstra and Cárdenas). Mapping of ancient sequences was first carried out using the default end-to-end option in Bowtie 2 v2.3.4^56^, to retain C→T/G→A misincorporations at terminal bases for damage pattern analysis. Sequences were then mapped to the concatenated reference genome a second time, after trimming 1 bp from each read end to remove putative ancient damage. All analyses except the ‘mapDamage’ analysis were done using these trimmed reads. Only reads of mapping quality >30 were retained for further analysis.

### Characterization of aDNA damage

We determined patterns of characteristic ancient DNA damage of reads mapping to each reference using mapDamage2.2.0^57^. For coral and Symbiodiniaceae, misincorporation plots were visually inspected for terminal base misincorporations. For microbial analysis, groups of reads mapping to each metagenome-assembled-genome were algorithmically sorted into modern and potentially ancient categories, using the following criteria: (1) the greatest C → T/G → A misincorporation rate at the terminal base of the read group, (2) 5’ C → T and 3’ G → A misincorporation rates higher at the terminal position than the average misincorporation rates +/− 2 standard deviations at this position, and (3) the C → T/G → A misincorporation rates must be less than the average misincorporation rates +/− 2 standard deviations at the next ten base positions. Read groups mapping to a given microbial species are scored by the number of criteria they meet. Misincorporation rate and read length distribution plots were also visually assessed for any potentially ancient read groups, but no discordance was found.

### Comparison of modern and ancient coral data

We compared ancient *A. palmata* data to modern data with two different methodologies. The first method follows the procedure in Kirch et al.^33^, considering ancient samples in principle component analysis (PCA) construction. In brief, we used ANGSD^58^ to create genotype likelihood files. The output files were then subset for positions covered in at least one of our four ancient samples. We then used PCAngsd^34^ to create PCAs of all samples at ancient covered positions. Genotype likelihoods from ANGSD were input to NGSAdmix^59^ to analyze admixture in our samples for K = 2 through K = 7. In parallel, we applied a method similar to Shinde et al^35^. Here, ancient samples are projected onto modern-day variation. To accomplish this, we determined biallelic single nucleotide variants (SNVs) in modern-day samples using ‘bcftools mpileup’^60^. Pseudohaplotype calls were then made using a combination of ‘samtools mpileup’ and sequenceTools^61^ ‘pileupCaller’. Finally, using EIGENSOFT ‘smartpca’^62^, PCAs were first constructed using data from modern samples, then ancient samples were projected into this space in order to reduce shrinkage effects on the ancient samples. To determine uncertainty about location of modern and ancient samples, we jackknifed our data about the 14 chromosome-level scaffolds plus two “pseudo-chromosomes” constructed from unplaced scaffolds in our *A. millepora* reference. We then projected the resulting “leave-one-out” samples into PCA space. All visualizations were completed in R with ggplot2^63^.

To calculate f4 statistics, we utilized ADMIXTOOLS^36^. Fifteen *Acropora millepora* whole genome sequencing samples were used as the outgroup population (from bioproject PRJNA593014^20^). We tested *f4(A. millepora* population, Ancient population; C population, D population) and *f4(A. millepora* population, B population; C population, D population) where B, C and D are all possible permutations of *A. palmata, A. cervicornis*, and *A. prolifera*. Significance was determined at |Z| ≥ 3.

### Microbial data analysis

Relative abundance of modern microbes was compared to ancient microbes by first restricting analysis solely to bacterial genomes present in at least one ancient sample. For each modern sample, counts of high quality (Q>30) reads mapping to each ancient MAG were calculated with samtools, then visualized in R with ggplot2. Additional analysis looked at overlapping presence of MAGs between modern and ancient samples and relative abundances of ancient-present MAGs in all samples.

To further confirm the specificity of mapped bacterial reads, we determined the taxonomic affiliation of all reads that mapped to bacterial genomes using KAIJU^64^ against the NCBI nr_euk database that includes all proteins belonging to viruses, archaea, bacteria, dinoflagellates, and other microbial eukaryotes (2019–06-25). The taxonomic level that best captured the genome taxonomy was reported (Figure S5).

## Results and Analysis

### Recovery and characterization of coral aDNA

**Figure 1.**
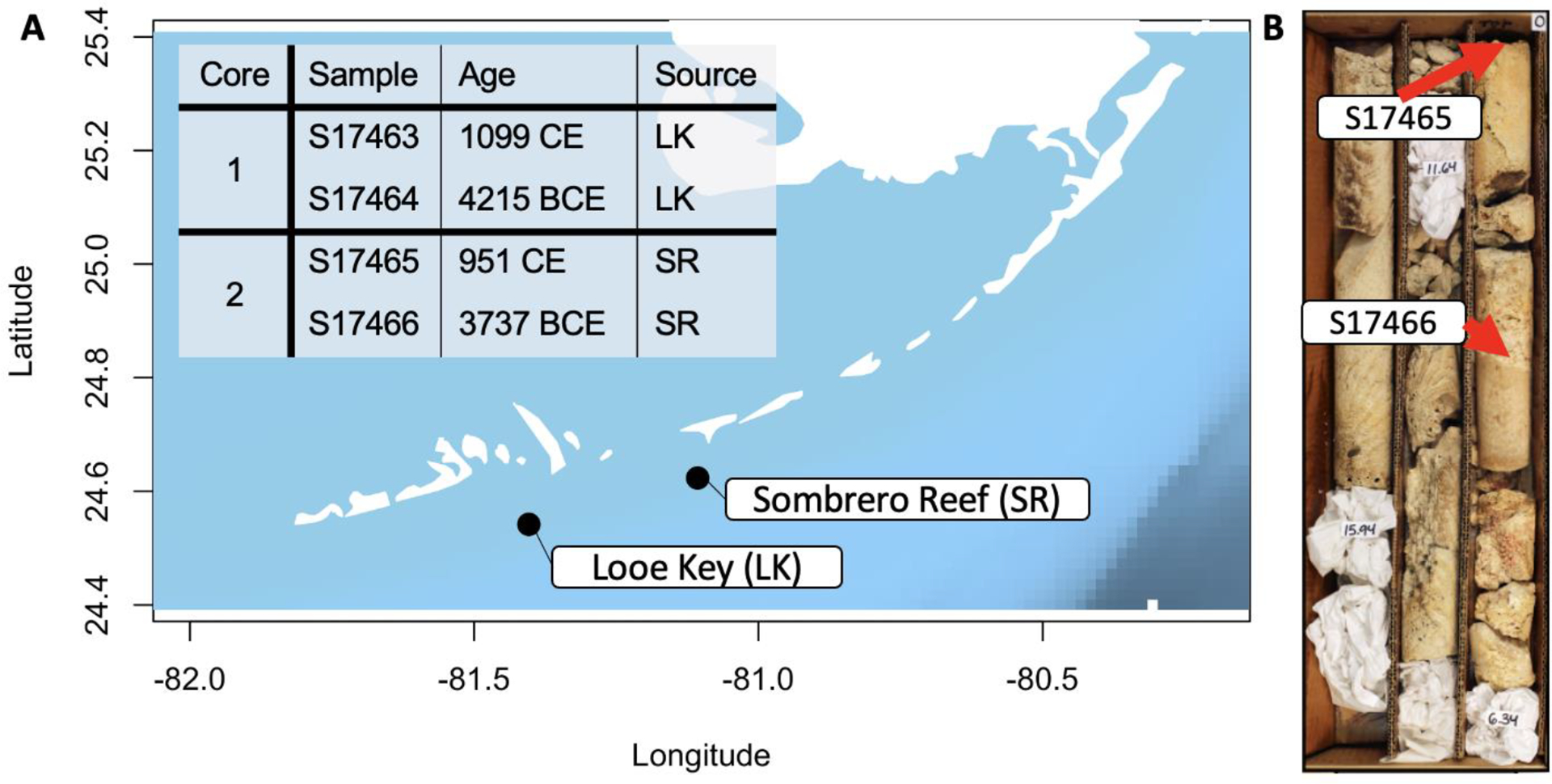
Samples originated from two geographically distinct cores dated between 4215 BCE and 951 CE. (A) Geographic source of the two cores used in this study. Inset gives the sample names associated with each core and the radiometrically determined age of each. (B) Photograph of Core 2 from USGS database. Red arrows indicate locations from within the core from which our samples were taken.

To understand ancient coral genetic diversity, we obtained samples from two Florida Keys reef cores in the U.S. Geological Survey (USGS) Core Archive housed at the USGS Coastal and Marine Science Center in St. Petersburg, Florida (Figure 1A–B; Table S1). Fossil samples were taken at different depths in the core from coral skeletons that were morphologically identified by LTT as *A. palmata* (Figure 1B)^22^. We prepared samples in dedicated clean rooms, extracted DNA^23,24^, and constructed libraries for Illumina sequencing^25^. The laboratory used to process these samples were from a human ancient DNA lab at Harvard Medical School, which had never processed coral samples previously. After partial UDG treatment of DNA libraries^26^, we shotgun sequenced samples on the Illumina HiSeq X10 platform.

We generated 58–70M Illumina read pairs for each sample (Table S2). To minimize issues associated with potential reference bias, we mapped to a concatenated reference consisting of *Acropora millepora* - a species equally diverged from all coral species found in the Florida Keys and the Caribbean. We also carried out all analysis mapping to *A. palmata* and observed similar results^27^. In addition, we mapped sequences to obligate endosymbionts *Symbiodinium, Breviolum, Cladocopium*, and *Durisdinium* (formerly clades A-D^28^), and 103 extant coral MAGs and cultured bacterial genomes (Cárdenas & Voolstra; associated data published with this project^29^). DNA recovered from ancient or historical material contains signatures of DNA damage over time that can be used to authenticate the origins of the genomic data collected. Characteristic aDNA molecules have short read-lengths and a high 5’ C→T or 3’ G→A misincorporation rate at the terminal ends of reads^30^.

**Figure 2.**
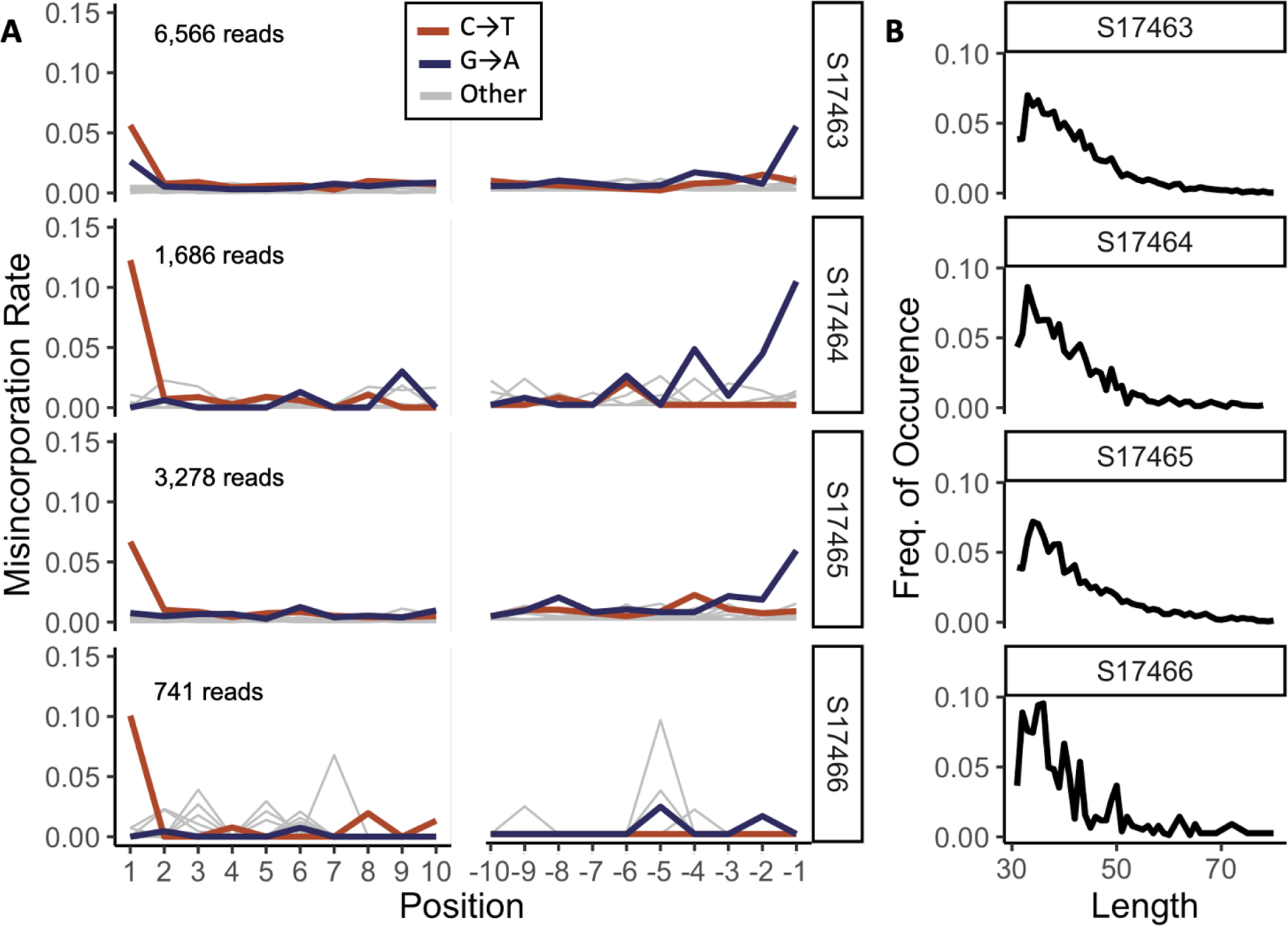
Terminal base misincorporation patterns and length distributions verify aDNA from coral host (A) Misincorporation rate of C→T and G→A substitutions across the first and last 10 bp of all mapped reads, given by sample. The expected pattern is seen across all samples, except at the 3’ end of S17466. This is likely an artifact of the low number of reads recovered from this sample. Inset numbers give mapped read pairs retrieved. (B) Read length distribution (base pairs) given by sample. All putatively ancient samples show similar distributions.

To examine these properties, after filtering for mapping quality (Q>30), we characterized aDNA damage patterns for reads mapping to each reference (Figure 2A–B). We identified between 30,615 and 293,984 putatively ancient reads from each fossil sample, mapping to the coral reference and ten of the MAGs (Table S2). Of those, between 741 and 6,566 mapped to our coral reference, with strong variation in data recovery dependent on sample age. This translated to 1.47 × 10^−6^ - 1.38 × 10^−5^ genomic coverage of the *A. millepora* reference. The coral-mapped proportion of each sample showed expected misincorporation patterns from UDG-half prepared libraries, as well as shortened length distributions (Figure 2A–B) in contrast to modern sequences (Figure S2). These patterns are in line with aDNA recovered from similarly dated carbonate shells of marine molluscs- a comparable preservation matrix^1^.

### Ancient Symbiodiniaceae reads were not present

Notably, we recovered few (an average of 243 per sample) *Symbiodiniaceae* reads prior to ancient filtering, and no samples showed an elevated C → T/G → A misincorporation rate at the terminal base pair (Table S2; Figure S1). It is likely that either symbiont DNA was not preserved within the reef matrix, or the endogenous content is so low we could not detect it using shotgun sequencing. Modern acroporid samples used in comparisons between present-day and ancient genetic variation showed no signatures of aDNA damage (Figure S2).

### Comparisons with extant acroporids

In order to place ancient samples in the context of modern-day variation, we retrieved modern-day whole genome sequencing data of *A. palmata, A. prolifera*, and *A. cervicornis* (N = 60) as well as microbial DNA samples associated with *A. palmata* (N = 177) from three recent studies^16,31,32^. These samples were collected from across the western Atlantic and overlapped with our study region in the Florida Keys (Table S3).

We employed two different methods to compare the ancient and modern acroporid data. These approaches account for uncertainty in ancient genotypes in different ways. The first analysis followed the method of Kirch *et al*.^33^, where PCAngsd was used to determine genotypes - restricted to genomic sites where single nucleotide variants (SNVs) were covered by at least one ancient sample. PCAngsd is designed for placing low-depth next-generation sequencing data using genotype likelihoods, which accounts for uncertainty in genotypes^34^. Notably, this method uses both ancient and modern samples to calculate principal component loadings.

In parallel, we carried out an analysis in the spirit of Shinde *et al*.^35^, which first used modern coral sequences to establish axes of variation upon which to project ancient samples. This approach potentially reduces bias which may make ancient samples artificially appear most related to each other (i.e., poorer data quality, damage patterns, etc.). As the number of reads we had available from the ancient coral sequences was low, we examined uncertainty in the PCA positions of these samples using a chromosome-level block jackknife.

**Figure 3.**
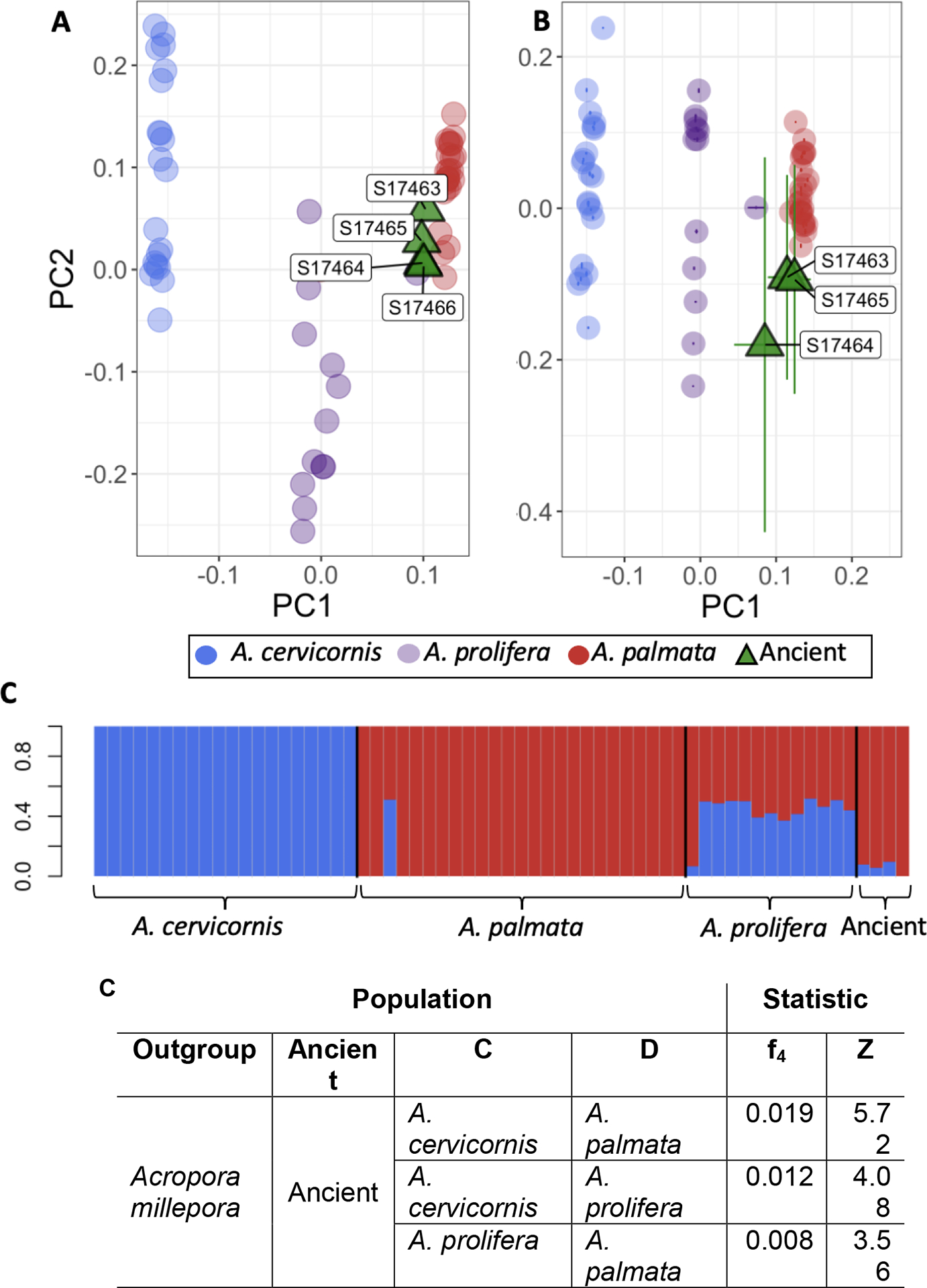
Comparison of coral aDNA with extant acroporids shows the predominance of *A. palmata* ancestry in ancient samples (A) PCAngsd-constructed PCA showing positions of ancient samples with respect to modern species clusters. Ancient samples (green triangles) group closest with *A. palmata*. (B) Projection-based PCA construction yielded qualitatively similar results, with the exception of the failure to place S17466. Bars about points represent PC-coordinate uncertainty calculated from chromosome jackknifing. (C) NGSadmix plot showing ancestry of different groups with K=2 (For K=3, see Figure S3). Each horizontal bar represents one individual from the labeled population. X-axis labels show the population each sample was assigned to morphologically prior to admixture analysis. Colored bars represent the proportion of ancestry from each group. (D) f_4_ statistics calculated for f_4_*(Outgroup, Ancient Individuals; C, D)*, where C, D are all possible combinations of *A. cervicornis*, *A. prolifera*, and *A. palmata*. Significance is determined by |Z| ≥ 3. Note, significant admixture is only found between ancient individuals and *A. palmata* or hybrid *A. prolifera*.

Both approaches yielded qualitatively similar results: the ancient DNA samples clustered closest with modern-day *A. palmata* in PCA space (though note the projection-based method could not place S17466 due to low coverage; Figure 3A–B). This confirms expectations, as ancient samples were morphologically identified as *A. palmata* (Table S1). Further, modern individuals clustered first by species (*A. palmata, A. cervicornis, A. prolifera)*, then by site (Figure S3). This recapitulates the results found by Kitchen et al.^16^, showing pseudo-haplotype data created in our projection-based approach accurately resolved known modern population clusters.

NGSAdmix analysis supports the results of the PCAs, confirming the assignment of our ancient samples to be mostly *A. palmata* (Figure 3C). Assuming two distinct genetic clusters (K=2), there is clear species delimitation between *A. palmata* and *A. cervicornis*, with evidence of mixed ancestry in the known hybrid *A. prolifera*. The small proportion of ancient samples attributed to *A. cervicornis* is likely noise in the data, disappearing for S17464 when K = 3, and presumably does not reflect ancestry from this congener (Figure S3). Together, the separation of ancient samples from *A. palmata* in PCA-space and small proportion of ancestry assigned to a second cluster show slight segregation between our ancient and modern samples (Figure 3A–B; Figure S4). This is potentially due to actual genetic differences accumulated over the last 1,000–6,000 years, or to artifacts arising from characteristics of aDNA (it also may reflect both phenomena together).

We next carried out formal analysis of ancestry and examined evidence (or lack thereof) of hybridization in our ancient samples through the application of f_4_ statistics^36^. First, we established a phylogeny comprising 4 modern coral samples found in the Florida Keys (namely *A. millepora*, *A. palmata, A. cervicornis* and *A. prolifera*). *A. prolifera* is a well-known hybrid, and indeed f_4_ statistics of the form f_4_*(A*, *B; C, D*) where A,B,C,D are varied across all permutations of the modern corals, confirm this hypothesis (Table S3). Following this, we aimed to determine which modern group our ancient samples shared the most alleles with by permuting f_4_*(Outgroup, Ancient; C, D)*, where *C* and *D* are all possible combinations of *A. palmata, A. prolifera*, and *A. cervicornis*. In all cases, the ancient samples only had significant ancestry from *A. palmata* or *A. prolifera* (*Z* ≥ *|3|*; Figure 3C). Given allele sharing with *A. prolifera* is only positive when compared against *A. cervicornis*, but not when compared to *A. palmata*, we inferred our samples to be most closely related to *A. palmata*.

### Identification of ancient microbes

Next, we examined reads from our sequencing data that mapped to our bacterial reference set. Of the 103 bacterial genomes used as a ‘search space’ in this study, a total of 10 had reads assigned from at least one ancient sample. These bacterial genomes corresponded mainly to bacterial populations associated with coral skeletons. Mapping largely occurred in moderate to highly conserved gene regions shared across members of the same phylum (Figure S5) and indicating the presence of representatives of the phyla Bacteroidota, Desulfobacterota, Myxococcota, Proteobacteria, and Spirochaetota in ancient samples. Only one bacterial representative from coral tissues was mapped by the ancient samples (R13_0) with enough taxonomic certainty to support the presence of members of the genus *Ruegeria* (Figure S5) in aDNA. Moreover, we found evidence for the presence of proteins involved in photosynthesis, nitrogen and carbon fixation, and sulfur oxidation among the ancient reads by means of mapping to the genes encoding the respective proteins.

### Comparison to modern microbiomes

**Fig 4.**
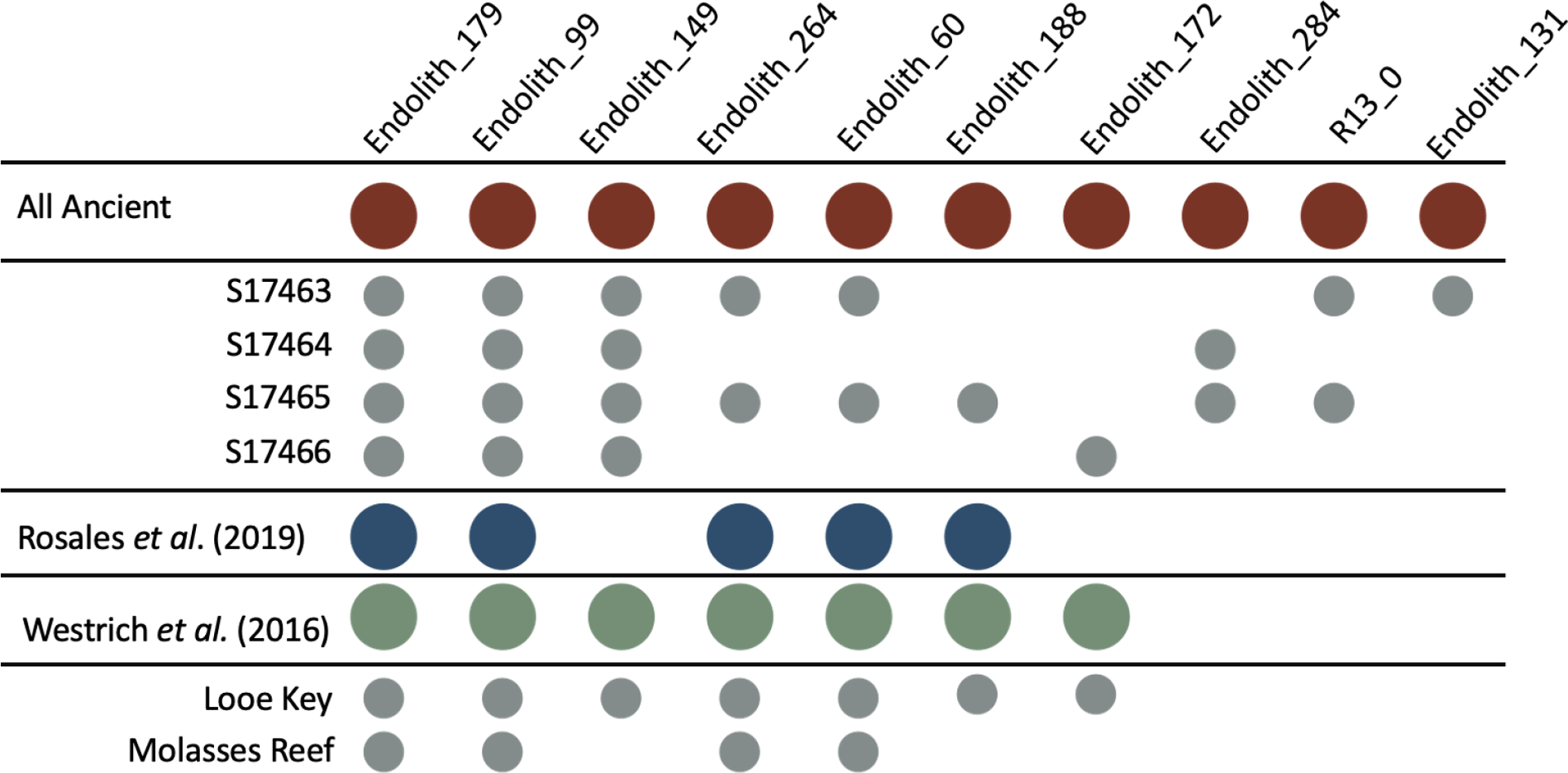
Ancient microbes overlap with modern day Florida Keys datasets. Plot of presence/absence of MAGs present in ancient samples against two recent studies from the Florida Keys. Grey dots represent subsets of samples from the study listed above them (i.e., Looe Key and Molasses Reef samples are both from Westrich *et al*.). Microbial richness was highest in the more modern samples (S17463 and S17465) and some microbes showed consistency across all groups (Endolith_179, Endolith_99, Endolith_149).

Additionally, ancient metagenomic data shared considerable overlap with modern microbial data. To determine if our ancient microbes could be found in modern-day coral holobionts, we compared our data against two acroporid studies from the Florida Keys^31,32^(Table S6). Of the 10 bacterial genomes to which the sequences from the ancient samples mapped, five were represented in both studies, and an additional two were present in data from Westrich *et al*.^31^ (Figure 4). Notably, microbe presence was variable within our ancient samples with the exception of Endolith_179, Endolith_99, and Endolith_149. More modern (S17463, S17465) aDNA samples showed higher microbial richness, though this may likely be due to preservation bias rather than reflecting a more diverse microbiome at that time point. Along the same vein, we are hesitant to draw conclusions about microbial absences (i.e., Endoltih_284, R13_0, and Endolith_131), as this may be attributable to sampling instead of function.

## Discussion

The presence of high confidence assigned sequence reads and expected damage patterns show it is possible to obtain ancient DNA from fossil coral fragments up to 6,000 years old. Ultimately, we only assigned ~0.01% of the original 50–70 million reads sequenced per sample to *A. millepora*. While this recovery rate is very low compared to typical data recovery rates in humans^37^, these values are in line with studies from 6,000–7,000 year old marine molluscs where only 0.02% - 1.7% of shotgun sequenced reads were retained with comparable genomic coverage^1^. In aDNA research applied to other organisms, target capture techniques have been used to increase target organism read retention or restrict analysis to known polymorphisms^38–40^ or enrich for damaged DNA molecules^41^. Specifically, Armbrecht et al.^38^ successfully applied hybridization-capture methodology to marine eukaryotes to greatly increase eukaryote and archaea DNA capture with respect to bacteria, while retaining patterns of aDNA damage. In the future, applications of capture technology to coral aDNA may similarly increase both the feasibility and efficiency of this method.

Further, the recovery of ancient microbial data allowed us to make preliminary comparisons between historical and modern microbiomes. It is well established that the coral holobiont can vary considerably over time and space, although certain coral species exhibit a remarkable extent of microbiome conservation^42–45^. Disparities in presence of microbes between ancient and modern samples may represent unique characteristics of the coral microbiome 1,000–6,000 years in the past which have not persisted to modern day. Notably, our ancient samples had the most similarity in presence and absence of ancient microbes with modern samples taken from the same reef (Looe Key). The least ancient MAGs were found in the data from Molasses reef - a reef geographically the furthest from the others. This result was not statistically significant (Fisher’s exact test; *p* = .3698), but it raises interesting questions about microbiome stability in both time and space. While not the primary goal of this study, future directions in coral aDNA could center on characterizing marine microbiomes across these dimensions.

Conversely, the presence of extant coral microbiome members in ancient samples reflects their (long-lasting) specificity and, by inference, their functional importance. Members of the phyla Myxococcota and Desulfobacterota are common associates of skeletal samples and presumed to play a role in sulfur cycling^26,27^, or nitrogen fixation if residing in the mucus^28^. Our results corroborated the presence of genes involved in sulfur cycling, and C and N fixation from these taxa in ancient samples, reaffirming their ecological niche in nutrient cycling, although their contribution to the coral holobiont remains unknown. Members of the Myxococcota have been linked to disease resistance in extant *A. palmata*^32^, and the presence of this bacterial group in ancient corals highlights the ancestry as well as consistency of beneficial microbial associations with putative insights into the evolution of coral holobiont health. Similarly, *Ruegeria* species are prevalent coral associates exhibiting a high taxonomic and functional diversity across coral compartments^46^, with putative beneficial roles for the holobiont in pathogen control^47,48^ and larval development^49,50^.

Long-term variation in marine microbes and eukaryotes using ancient DNA has previously yielded exciting results about marine community composition at distant timescales. For example, Ambrechet et al.^34^ revealed dynamics of harmful algal blooms using marine sediment cores up to 9,000 years in the past. In a similar vein, Cabrera et al.^35^ explored variability of coral species dominance and their associated algae over the last 750 years. Because coral microbiomes generate specific microbial fingerprints depending on the host physiological state or environmental condition, extending coral reef aDNA studies to include their associated microbial communities would provide a holistic perspective of the evolution and role of microbial associations in coral health.

In the western Atlantic, gaining insight into evolutionary trajectories of coral reefs is increasingly important as extant corals are declining at an unprecedented rate. Since the 1980s, coral cover has declined by 90% in the region and reef-building corals like *A. palmata* have been hit particularly hard^5,6^. The ability to assess changes in genetic variation of key coral taxa in response to past stressors will enable better prediction of extant corals’ responses to continued environmental disturbances in the future. Using ancient DNA, it is possible to investigate these past changes directly. Has genetic variation in *A. palmata* declined in recent history? Were these populations able to adapt to significant population pressures in the past? Do we see the same population structure between locations in ancient *A. palmata* as we do modern? Can we calculate genetic connectivity between ancient reefs, and, if so, what might this reveal about ocean dynamics? Through coral aDNA we can gain a “window into the past” to evaluate the evolutionary history of these populations.

Overall, this study serves as a promising proof-of-concept to root future applications of aDNA in coral reef biology. Due to low ancient read recovery rate, future coral aDNA studies will have to leverage target enrichment technologies such as hybridization capture to make broader scale aDNA surveys feasible. However, we demonstrate that it is possible to extract nuclear DNA from corals up to 6,000 years old and place these data in the context of modern-day genetic variation, both for the coral host and associated microbes. Future studies will clarify the separation observed between ancient and modern populations, how coral hosts and their associated microbes change over time, and the presence of evolutionary constraints that may limit their adaptive potential. Building on this work could allow for a holistic perspective on coral ecology over millennial timescales and beyond.

## Supplementary Material

Supplementary Material

## Data Availability

All DNA sequences from fossil coral cores can be found at BioProject #PRJNA757238. Coral holobiont endolith bacterial genomes can be found at BioProject #PRJNA757245. Code used for data processing as well as protocols used for library preparations can be found at https://github.com/cb-scott/coral-aDNA.

## References

[1] Der Sarkissian C Unveiling the Ecological Applications of Ancient DNA From Mollusk Shells. Front. Ecol. Evol 8, 1–21 (2020).

[2] Padró J, Lambertucci SA, Perrig PL & Pauli JN Andean and California condors possess dissimilar genetic composition but exhibit similar demographic histories. Ecol. Evol 1–11 (2020) doi:10.1002/ece3.6887. PMC7713948 33304512

[3] Orlando L Recalibrating Equus evolution using the genome sequence of an early Middle Pleistocene horse. Nature 499, 74–78 (2013). 23803765 10.1038/nature12323

[4] Narasimhan VM The formation of human populations in South and Central Asia. Science 365, (2019). 10.1126/science.aat7487 PMC6822619 31488661

[5] Gardner TA, Côté IM, Gill JA, Grant A & Watkinson AR Long-term region-wide declines in Caribbean corals. Science 301, 958–960 (2003). 12869698 10.1126/science.1086050

[6] Kuffner IB Plasticity in skeletal characteristics of nursery-raised staghorn coral, Acropora cervicornis. Coral Reefs 36, 679–684 (2017).

[7] Glasl B Microbial indicators of environmental perturbations in coral reef ecosystems. Microbiome 7, 1–13 (2019). 31227022 10.1186/s40168-019-0705-7 PMC6588946

[8] Hugerth LW Metagenome-assembled genomes uncover a global brackish microbiome. Genome Biol. 16, 279 (2015). 26667648 10.1186/s13059-015-0834-7 PMC4699468

[9] Parks DH Recovery of nearly 8,000 metagenome-assembled genomes substantially expands the tree of life. Nat. Microbiol 2, 1533–1542 (2017). 28894102 10.1038/s41564-017-0012-7

[10] Tully BJ, Graham ED & Heidelberg JF The reconstruction of 2,631 draft metagenome-assembled genomes from the global oceans. Sci. Data 5, 170203 (2018). 29337314 10.1038/sdata.2017.203 PMC5769542

[11] Wilkins LGE, Ettinger CL, Jospin G & Eisen JA Metagenome-assembled genomes provide new insight into the microbial diversity of two thermal pools in Kamchatka, Russia. Sci. Rep 9, 3059 (2019). 30816235 10.1038/s41598-019-39576-6 PMC6395817

[12] Cárdenas A Excess labile carbon promotes the expression of virulence factors in coral reef bacterioplankton. ISME J. 12, 59–76 (2018). 28895945 10.1038/ismej.2017.142 PMC5739002

[13] Shibl AA Diatom modulation of select bacteria through use of two unique secondary metabolites. Proc. Natl. Acad. Sci. U. S. A 117, 27445–27455 (2020). 33067398 10.1073/pnas.2012088117 PMC7959551

[14] Robbins SJ A genomic view of the reef-building coral Porites lutea and its microbial symbionts. Nat. Microbiol 4, 2090–2100 (2019). 31548681 10.1038/s41564-019-0532-4

[15] Wibowo MC Reconstruction of ancient microbial genomes from the human gut. Nature 594, 234–239 (2021). 33981035 10.1038/s41586-021-03532-0 PMC8189908

[16] Kitchen SA Genomic variants among threatened Acropora corals. G3 Genes Genomes Genet. 9, 1633–1646 (2019). 10.1534/g3.119.400125 PMC6505131 30914426

[17] Ayre DJ & Hughes TP Genotypic Diversity and Gene Flow in Brooding and Spawning Corals Along the Great Barrier Reef, Australia. Evolution 54, 1590–1605 (2000). 11108587 10.1111/j.0014-3820.2000.tb00704.x

[18] Cunning R Census of heat tolerance among Florida’s threatened staghorn corals finds resilient individuals throughout existing nursery populations. Proc. R. Soc. B Biol. Sci 288, 20211613 (2021). 10.1098/rspb.2021.1613 PMC8527199 34666521

[19] Richards ZT, Oppen M. J. H. van, Wallace CC, Willis BL & Miller DJ Some Rare Indo-Pacific Coral Species Are Probable Hybrids. PLOS ONE 3, e3240 (2008). 18813338 10.1371/journal.pone.0003240 PMC2531234

[20] Fuller ZL Population genetics of the coral Acropora millepora: Toward genomic prediction of bleaching. Science 369, eaba4674 (2020). 32675347 10.1126/science.aba4674

[21] Matz MV, Treml EA, Aglyamova GV & Bay LK Potential and limits for rapid genetic adaptation to warming in a Great Barrier Reef coral. PLoS Genet. 14, 1–19 (2018). 10.1371/journal.pgen.1007220 PMC5908067 29672529

[22] Toth LT The unprecedented loss of Florida’s reef-building corals and the emergence of a novel coral-reef assemblage. Ecology 100, e02781 (2019). 31170313 10.1002/ecy.2781 PMC6851685

[23] Dabney J Complete mitochondrial genome sequence of a Middle Pleistocene cave bear reconstructed from ultrashort DNA fragments. Proc. Natl. Acad. Sci 110, 15758–15763 (2013). 24019490 10.1073/pnas.1314445110 PMC3785785

[24] Korlević P Reducing microbial and human contamination in DNA extractions from ancient bones and teeth. BioTechniques 59, 87–93 (2015). 26260087 10.2144/000114320

[25] Meyer M A High-Coverage Genome Sequence from an Archaic Denisovan Individual. Science 338, 222–226 (2012). 22936568 10.1126/science.1224344 PMC3617501

[26] Rohland N, Harney E, Mallick S, Nordenfelt S & Reich D Partial uracil–DNA–glycosylase treatment for screening of ancient DNA. Philos. Trans. R. Soc. B Biol. Sci 370, 20130624 (2015). 10.1098/rstb.2013.0624 PMC4275898 25487342

[27] Günther T & Nettelblad C The presence and impact of reference bias on population genomic studies of prehistoric human populations. PLOS Genet. 15, e1008302 (2019). 31348818 10.1371/journal.pgen.1008302 PMC6685638

[28] LaJeunesse TC Systematic Revision of Symbiodiniaceae Highlights the Antiquity and Diversity of Coral Endosymbionts. Curr. Biol 28, 2570–2580.e6 (2018). 30100341 10.1016/j.cub.2018.07.008

[29] Cardénas A & Voolstra CR 75 Coral Endolith Bacterial Genomes (MAGs) from Red Sea corals Goniastrea edwardsi and Porites lutea. (2021) doi:10.5281/zenodo.5606932.

[30] Briggs AW Patterns of damage in genomic DNA sequences from a Neandertal. Proc. Natl. Acad. Sci. U. S. A 104, 14616–14621 (2007). 17715061 10.1073/pnas.0704665104 PMC1976210

[31] Westrich JR Saharan dust nutrients promote Vibrio bloom formation in marine surface waters. Proc. Natl. Acad. Sci 113, 5964–5969 (2016). 27162369 10.1073/pnas.1518080113 PMC4889353

[32] Rosales SM Microbiome differences in disease-resistant vs. susceptible Acropora corals subjected to disease challenge assays. Sci. Rep 9, 18279 (2019). 31797896 10.1038/s41598-019-54855-y PMC6892807

[33] Kirch M, Romundset A, Gilbert MTP, Jones FC & Foote AD Ancient and modern stickleback genomes reveal the demographic constraints on adaptation. Curr. Biol 31, 2027–2036.e8 (2021). 33705715 10.1016/j.cub.2021.02.027

[34] Meisner J & Albrechtsen A Inferring Population Structure and Admixture Proportions in Low-Depth NGS Data. Genetics 210, 719–731 (2018). 30131346 10.1534/genetics.118.301336 PMC6216594

[35] Shinde V An Ancient Harappan Genome Lacks Ancestry from Steppe Pastoralists or Iranian Farmers. Cell 179, 729–735.e10 (2019). 31495572 10.1016/j.cell.2019.08.048 PMC6800651

[36] Patterson N Ancient Admixture in Human History. Genetics 192, 1065–1093 (2012). 22960212 10.1534/genetics.112.145037 PMC3522152

[37] Skoglund P Origins and genetic legacy of Neolithic farmers and hunter-gatherers in Europe. Science 336, 466–469 (2012). 22539720 10.1126/science.1216304

[38] Armbrecht L, Hallegraeff G, Bolch CJS, Woodward C & Cooper A Hybridisation capture allows DNA damage analysis of ancient marine eukaryotes. Sci. Rep 11, 3220 (2021). 33547359 10.1038/s41598-021-82578-6 PMC7864908

[39] Haak W Massive migration from the steppe was a source for Indo-European languages in Europe. Nature 522, 207–211 (2015). 25731166 10.1038/nature14317 PMC5048219

[40] Fu Q An early modern human from Romania with a recent Neanderthal ancestor. Nature 524, 216–219 (2015). 26098372 10.1038/nature14558 PMC4537386

[41] Gansauge M-T & Meyer M Selective enrichment of damaged DNA molecules for ancient genome sequencing. Genome Res. 24, 1543–1549 (2014). 25081630 10.1101/gr.174201.114 PMC4158764

[42] Guppy R & Bythell JC Environmental effects on bacterial diversity in the surface mucus layer of the reef coral Montastraea faveolata. Mar. Ecol. Prog. Ser 328, 133–142 (2006).

[43] Klaus JS, Frias-Lopez J, Bonheyo GT, Heikoop JM & Fouke BW Bacterial communities inhabiting the healthy tissues of two Caribbeanreef corals: interspecific and spatial variation. Coral Reefs 24, 129–137 (2005).

[44] Morrow K, Moss A, Chadwick N & Liles M Bacterial Associates of Two Caribbean Coral Species Reveal Species-Specific Distribution and Geographic Variability. Appl. Environ. Micribiology 78, 6438–6449 (2012). 10.1128/AEM.01162-12 PMC3426691 22773636

[45] Neave MJ Differential specificity between closely related corals and abundant Endozoicomonas endosymbionts across global scales. ISME J. 11, 186–200 (2017). 27392086 10.1038/ismej.2016.95 PMC5335547

[46] Luo D Population differentiation of Rhodobacteraceae along with coral compartments. ISME J. 1–17 (2021) doi:10.1038/s41396-021-01009-6. PMC8528864 34017056

[47] Miura N Ruegeria sp. Strains Isolated from the Reef-Building Coral Galaxea fascicularis Inhibit Growth of the Temperature-Dependent Pathogen Vibrio coralliilyticus. Mar. Biotechnol. N. Y. N 21, 1–8 (2019). 10.1007/s10126-018-9853-1 30194504

[48] Garren M A bacterial pathogen uses dimethylsulfoniopropionate as a cue to target heat-stressed corals. ISME J. 8, 999–1007 (2014). 24335830 10.1038/ismej.2013.210 PMC3996689

[49] Apprill A, Marlow HQ, Martindale MQ & Rappé MS The onset of microbial associations in the coral Pocillopora meandrina. ISME J. 3, 685–699 (2009). 19242535 10.1038/ismej.2009.3

[50] Freire I, Gutner-Hoch E, Muras A, Benayahu Y & Otero A The effect of bacteria on planula-larvae settlement and metamorphosis in the octocoral Rhytisma fulvum fulvum. PLOS ONE 14, e0223214 (2019). 31568517 10.1371/journal.pone.0223214 PMC6768449

[51] Shinn EA, Hudson JH, Halley RB & Lidz BH Topographic control and accumulation rate of some Holocene coral reefs: south Florida and Dry Tortugas. 2: Geology, 1–7 (1977).

[52] Toth L, Stathakopoulos A & Kuffner I Descriptive Core Logs, Core Photographs, Radiocarbon Ages, and Accretion Data from Holocene Reef Cores Collected Throughout the Florida Keys Reef Tract. U.S. Geological Survey data release (2018) doi:10.5066/F7NV9HJX.

[53] Rohland N, Glocke I, Aximu-Petri A & Meyer M Extraction of highly degraded DNA from ancient bones, teeth and sediments for high-throughput sequencing. Nat. Protoc 13, 2447–2461 (2018). 30323185 10.1038/s41596-018-0050-5

[54] Shoguchi E Two divergent Symbiodinium genomes reveal conservation of a gene cluster for sunscreen biosynthesis and recently lost genes. BMC Genomics 19, 458 (2018). 29898658 10.1186/s12864-018-4857-9 PMC6001144

[55] Shoguchi E Draft assembly of the Symbiodinium minutum nuclear genome reveals dinoflagellate gene structure. Curr. Biol. CB 23, 1399–1408 (2013). 23850284 10.1016/j.cub.2013.05.062

[56] Langmead B & Salzberg SL Fast gapped-read alignment with Bowtie 2. Nat. Methods 9, 357–359 (2012). 22388286 10.1038/nmeth.1923 PMC3322381

[57] Jónsson H, Ginolhac A, Schubert M, Johnson PLF & Orlando L MapDamage2.0: Fast approximate Bayesian estimates of ancient DNA damage parameters. Bioinformatics 29, 1682–1684 (2013). 23613487 10.1093/bioinformatics/btt193 PMC3694634

[58] Korneliussen TS, Albrechtsen A & Nielsen R ANGSD: Analysis of Next Generation Sequencing Data. BMC Bioinformatics 15, 356 (2014). 25420514 10.1186/s12859-014-0356-4 PMC4248462

[59] Skotte L, Korneliussen TS & Albrechtsen A Estimating Individual Admixture Proportions from Next Generation Sequencing Data. Genetics (2013). 10.1534/genetics.113.154138 PMC3813857 24026093

[60] Li H The Sequence Alignment/Map format and SAMtools. Bioinforma. Oxf. Engl 25, 2078–2079 (2009). 10.1093/bioinformatics/btp352 PMC2723002 19505943

[61] Schiffels S sequenceTools. (2015).

[62] Galinsky KJ Fast Principal-Component Analysis Reveals Convergent Evolution of ADH1B in Europe and East Asia. Am. J. Hum. Genet 98, 456–472 (2016). 26924531 10.1016/j.ajhg.2015.12.022 PMC4827102

[63] Wickham H ggplot2: Elegant Graphics for Data Analysis. (Springer-Verlag, 2009). doi:10.1007/978-0-387-98141-3.

[64] Menzel P, Ng KL & Krogh A Fast and sensitive taxonomic classification for metagenomics with Kaiju. Nat. Commun 7, 11257 (2016). 27071849 10.1038/ncomms11257 PMC4833860

