# Supplementary material for: Millennia-old coral holobiont DNA provides insight into future adaptive trajectories
Source: Mol Ecol. Author manuscript; Available in PMC 2024 Nov 22. (PMC11584277; doi:10.1111/mec.16642)
Supplement: Supplementary Material [file NIHMS2035083-supplement-Supplementary_Material.docx]

**Supplemental Information & Figures**

**Table S1***.* **Age, species, and geography assignment by sample**

Information on sample IDs, ages, species and location (Toth et al., 2018). See also **Figure 1**. Age uncertainty given as two standard deviations.

| Core | Sample | Age | Age Uncertainty | Species | Location |
| --- | --- | --- | --- | --- | --- |
| 1 | S17463 | 1099 CE | 954-1267 CE | *A. palmata* | Looe Key, Florida Keys |
|  | S17464 | 4215 BCE | 4312-4096 BCE | *A. palmata* | Looe Key, Florida Keys |
| 2 | S17465 | 951 CE | 849-1036 CE | *A. palmata* | Sombrero Reef, Florida Keys |
|  | S17466 | 3737 BCE | 3860-3640 BCE | *A. palmata* | Sombrero Reef, Florida Keys |

**Table S2. Reads mapped by scaffold and proportion identified as putatively ancient.** Table giving reads mapped to each part of concatenated reference by sample, after filtering for mapping quality (Q>30). Bolded scaffolds marked with a (*) indicate a reference to which at least one sample showed characteristic aDNA patterns. Bolded and italicized table entries indicate which samples were identified as putatively ancient for a given reference

| Scaffold | Number Read Mapped by Sample | | | |
| --- | --- | --- | --- | --- |
|  | S17463 | S17464 | S17465 | S17466 |
| ***A. millepora **** | ***6566*** | ***1686*** | ***3278*** | ***741*** |
| *Symbiodinium* | 189 | 79 | 277 | 1013 |
| *Breviolum* | 55 | 43 | 68 | 1457 |
| *Cladicopium* | 95 | 60 | 70 | 308 |
| *Durisdinium* | 158 | 26 | 132 | 215 |
| A19_0 | 1171 | 1652 | 557 | 645 |
| A3_1 | 74032 | 3016 | 47032 | 26590 |
| A6_0 | 7804 | 36191 | 250 | 826 |
| A7_0 | 107605 | 4639523 | 35161 | 858351 |
| Endolith_101 | 201 | 34 | 60 | 38 |
| Endolith_106 | 26 | 2 | 99 | 102 |
| Endolith_120 | 94 | 55 | 337 | 651 |
| Endolith_124 | 17 | 14 | 35 | 48 |
| Endolith_130 | 1719 | 1316 | 1472 | 870 |
| **Endolith_131 *** | ***1613*** | 25 | 125 | 37 |
| Endolith_138 | 82 | 66 | 74 | 103 |
| Endolith_141 | 169 | 22 | 35 | 49 |
| Endolith_144 | 433 | 235 | 1103 | 1803 |
| Endolith_147 | 9 | 8 | 169 | 57 |
| **Endolith_149 *** | ***12605*** | ***8153*** | ***43730*** | ***12736*** |
| Endolith_159 | 858 | 622 | 580 | 730 |
| Endolith_160 | 67 | 39 | 102 | 48 |
| Endolith_161 | 30 | 9 | 120 | 34 |
| Endolith_172 | 1440 | 1694 | 1460 | 2205 |
| Endolith_175 | 40 | 15 | 466 | 128 |
| **Endolith_179 *** | ***137231*** | ***1998*** | ***38076*** | ***20791*** |
| Endolith_183 | 38 | 34 | 24 | 16 |
| Endolith_184 | 79 | 19 | 63 | 44 |
| **Endolith_188 *** | 719 | 218 | ***1313*** | 187 |
| Endolith_189 | 44 | 7 | 127 | 57 |
| Endolith_191 | 186 | 33 | 415 | 25 |
| Endolith_200 | 228 | 29 | 53 | 38 |
| Endolith_202 | 52 | 27 | 1677 | 37 |
| Endolith_217 | 19762 | 51335 | 264391 | 2330836 |
| Endolith_218 | 615 | 191 | 256 | 452 |
| Endolith_221 | 43 | 32 | 89 | 7 |
| Endolith_222 | 173 | 13 | 85 | 31 |
| Endolith_224 | 9717 | 4650 | 94143 | 94792 |
| Endolith_232 | 1002 | 199 | 261 | 299 |
| Endolith_237 | 914 | 778 | 393 | 351 |
| Endolith_240 | 207 | 24 | 224 | 72 |
| Endolith_245 | 24615 | 133801 | 7553579 | 7321526 |
| Endolith_246 | 701 | 182 | 248 | 97 |
| Endolith_254 | 58 | 16 | 64 | 29 |
| Endolith_259 | 57 | 54 | 63 | 64 |
| **Endolith_264 *** | ***43175*** | 376 | ***13875*** | 225 |
| Endolith_271 | 164 | 25 | 116 | 0 |
| Endolith_272 | 97 | 561 | 189 | 37 |
| Endolith_277 | 140 | 74 | 197 | 44 |
| Endolith_280 | 92 | 66 | 127 | 27 |
| Endolith_282 | 668 | 142 | 276 | 178 |
| Endolith_283 | 543 | 643 | 357 | 88 |
| **Endolith_284 *** | 405 | ***2294*** | ***2603*** | 208 |
| Endolith_290 | 45114 | 3608 | 5188 | 14492 |
| Endolith_291 | 550 | 529 | 393 | 823 |
| Endolith_294 | 29 | 6 | 217 | 22 |
| Endolith_296 | 498 | 130 | 167 | 235 |
| Endolith_298 | 114956 | 14712 | 4913 | 12538 |
| Endolith_299 | 201 | 33 | 113 | 85 |
| Endolith_303 | 582 | 461 | 207 | 160 |
| Endolith_311 | 31 | 17 | 27 | 85 |
| Endolith_313 | 532 | 280 | 149 | 50 |
| Endolith_318 | 621 | 24 | 89 | 119 |
| Endolith_339 | 131 | 11 | 98 | 26 |
| Endolith_343 | 16 | 37 | 74 | 19 |
| Endolith_352 | 130 | 10 | 88 | 29 |
| Endolith_353 | 1271 | 367 | 437 | 275 |
| Endolith_374 | 28 | 17 | 3 | 4 |
| Endolith_37 | 191 | 182 | 182 | 77 |
| Endolith_395 | 14 | 7 | 12 | 0 |
| Endolith_422 | 552 | 151 | 184 | 660 |
| Endolith_430 | 117 | 3 | 177 | 7 |
| Endolith_55 | 335 | 114 | 183 | 44 |
| Endolith_56 | 58 | 50 | 31 | 189 |
| **Endolith_60 *** | ***6563*** | 515 | ***14969*** | 253 |
| Endolith_64 | 28 | 0 | 41 | 19 |
| Endolith_66 | 183 | 81 | 155 | 19 |
| Endolith_67 | 67 | 26 | 141 | 13 |
| Endolith_86 | 162 | 9 | 33 | 22 |
| Endolith_87 | 54 | 13 | 108 | 137 |
| Endolith_89 | 1458 | 1414 | 2388 | 760 |
| Endolith_91 | 150 | 33 | 235 | 61 |
| Endolith_98 | 1414 | 198 | 606 | 1794 |
| **Endolith_99 *** | ***83779*** | ***16484*** | ***47652*** | ***6026*** |
| G20_0 | 779 | 1237 | 5419 | 4995 |
| G21_0 | 6065 | 180440 | 4115 | 1669 |
| G2_1 | 204 | 54 | 69 | 265 |
| G2_2 | 316 | 34 | 41 | 137 |
| R10_1 | 0 | 0 | 0 | 0 |
| R10_2 | 0 | 0 | 0 | 0 |
| R11_0 | 99 | 516 | 660 | 1249 |
| R12_1 | 0 | 0 | 0 | 0 |
| R12_2 | 1 | 0 | 0 | 0 |
| **R13_0 *** | ***2452*** | 1511 | ***2396*** | 2494 |
| R14_0 | 329 | 9199 | 9461 | 16702 |
| R15_0 | 535 | 36617 | 15830 | 21483 |
| R16_0 | 539 | 2902 | 3449 | 5156 |
| R17_0 | 383 | 4118 | 7217 | 10953 |
| R18_1 | 348 | 210926 | 50262 | 39309 |
| R18_2 | 417 | 202013 | 49272 | 67092 |
| R4_1 | 137 | 92 | 131 | 56 |
| R4_2 | 110 | 1276 | 501 | 644 |
| R5_0 | 922 | 1390 | 988 | 1122 |
| R8_1 | 0 | 2 | 43 | 25 |
| R8_2 | 0 | 0 | 0 | 0 |
| R9_1 | 79 | 234 | 278 | 415 |
| R9_2 | 65 | 292 | 235 | 555 |
| R9_3 | 61 | 295 | 265 | 509 |
| **Total Mapped:** | 732459 | 5585076 | 8339968 | 10894216 |
| **Reads Sequenced:** | 57903514 | 56354049 | 72336825 | 60753563 |
| **Percent Mapped:** | 0.01265 | 0.099107 | 0.115294 | 0.179318 |
| **Putatively Ancient:** | 293984 | 30615 | 167892 | 42499 |
| **Proportion Ancient/ Total Mapped** | 0.40136581 | 0.00548157 | 0.02013101 | 0.00390106 |

**Table S3. f_4_ statistics for modern day acroporids.** Both *A. palmata* and *A. cervicornis* are more related to known hybrid *A. prolifera* than each other.

| **Outgroup** | **B** | **C** | **D** | **f_4_** | **Z** |
| --- | --- | --- | --- | --- | --- |
| *Acropora millepora* | *A. palmata* | *A. cervicornis* | *A. prolifera* | 0.0119 | 27.05 |
| *Acropora millepora* | *A. cervicornis* | *A. palmata* | *A. prolifera* | 0.0077 | 23.36 |
| *Acropora millepora* | *A. prolifera* | *A. cervicornis* | *A. palmata* | 0.0046 | 19.52 |

**Table S4. Details on comparison data sets used.** Citations, details, and BioProject Accession numbers for all modern comparison data sourced for this study. Note there is more data associated with Westrich *et al.* than initially published, not all of which was used in comparison for this study.

| Study | Accession | Data Type | # Samples | Sample Source | Used? |
| --- | --- | --- | --- | --- | --- |
| Kitchen *et al.* (2019) | PRJNA473816 | Whole-genome sequencing | 21 | *A. cervicornis* | Yes |
|  |  |  | 26 | *A. palmata* | Yes |
|  |  |  | 13 | *A. prolifera* | Yes |
| Rosales *et al.* (2019) | PRJNA546259 | 16sRNA sequencing | 96 | *A. cervicornis* and *A. palmata*  tissue | Yes |
| Westrich *et al.* (2016) | PRJNA299413 | 16sRNA sequencing | 81 | *A. palmata* mucus | Yes |
|  |  |  | 44 | Water sample | No |


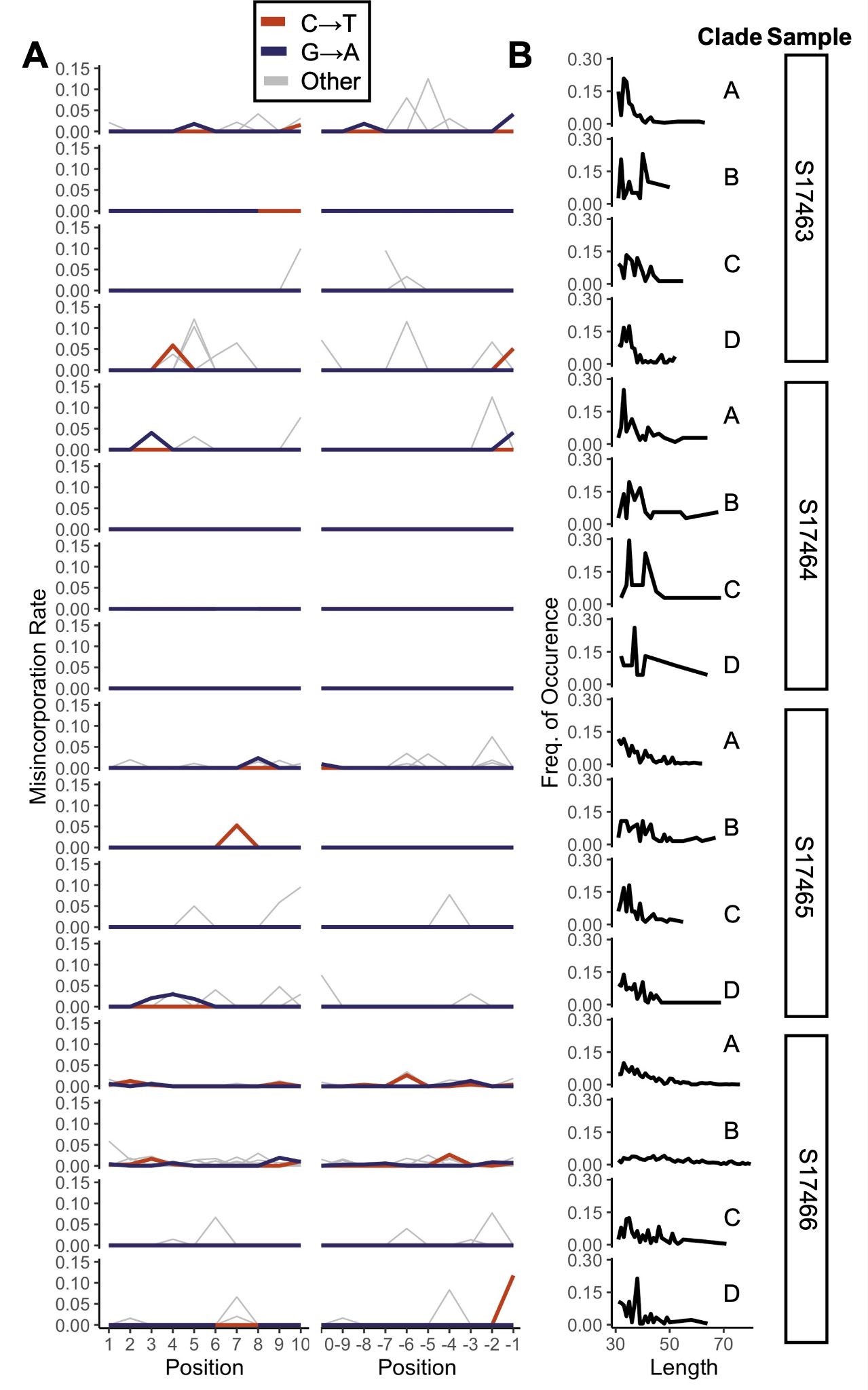


**Figure S1. Misincorporation rates at the terminal ends of reads do not show characteristic aDNA patterns in *Symbiodineacea*.** Clade refers to species of symbiont (formerly clades A-D), and sample refers to the source. (A) Misincorporation plots for each sample/symbiont combination. Note that we do not observe an elevated C→T and G→A misincorporation pattern at the terminal bases in any sample. This is especially clear when compared to **Figure 2.** (B) Read length distribution for each sample and symbiont combination. The sample with the most symbiont reads (S17466) does not show the same length distribution as expected from aDNA.


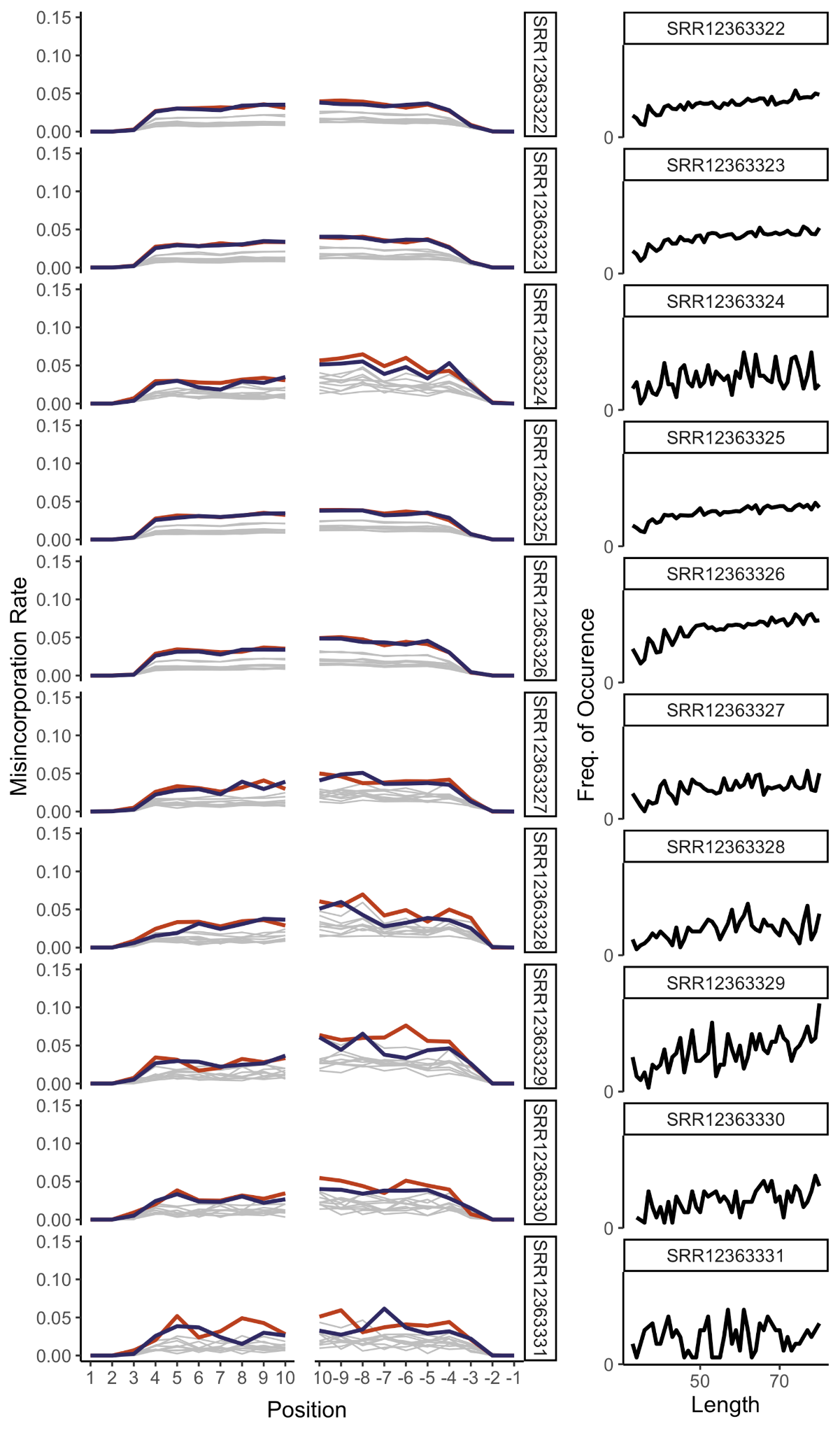


**Figure S2. Modern acroporid data does not show the same misincorporation rate and length distribution patterns as ancient coral.** (A) Misincorporation plots broken down by sample for ten modern files*.* This pattern holds for all modern acroporid data. (B) Read length distributions for the corresponding sample. Low misincorporation rate at terminal ends is an artifact of read mapping. However, these bases *were not* soft clipped from the modern files.


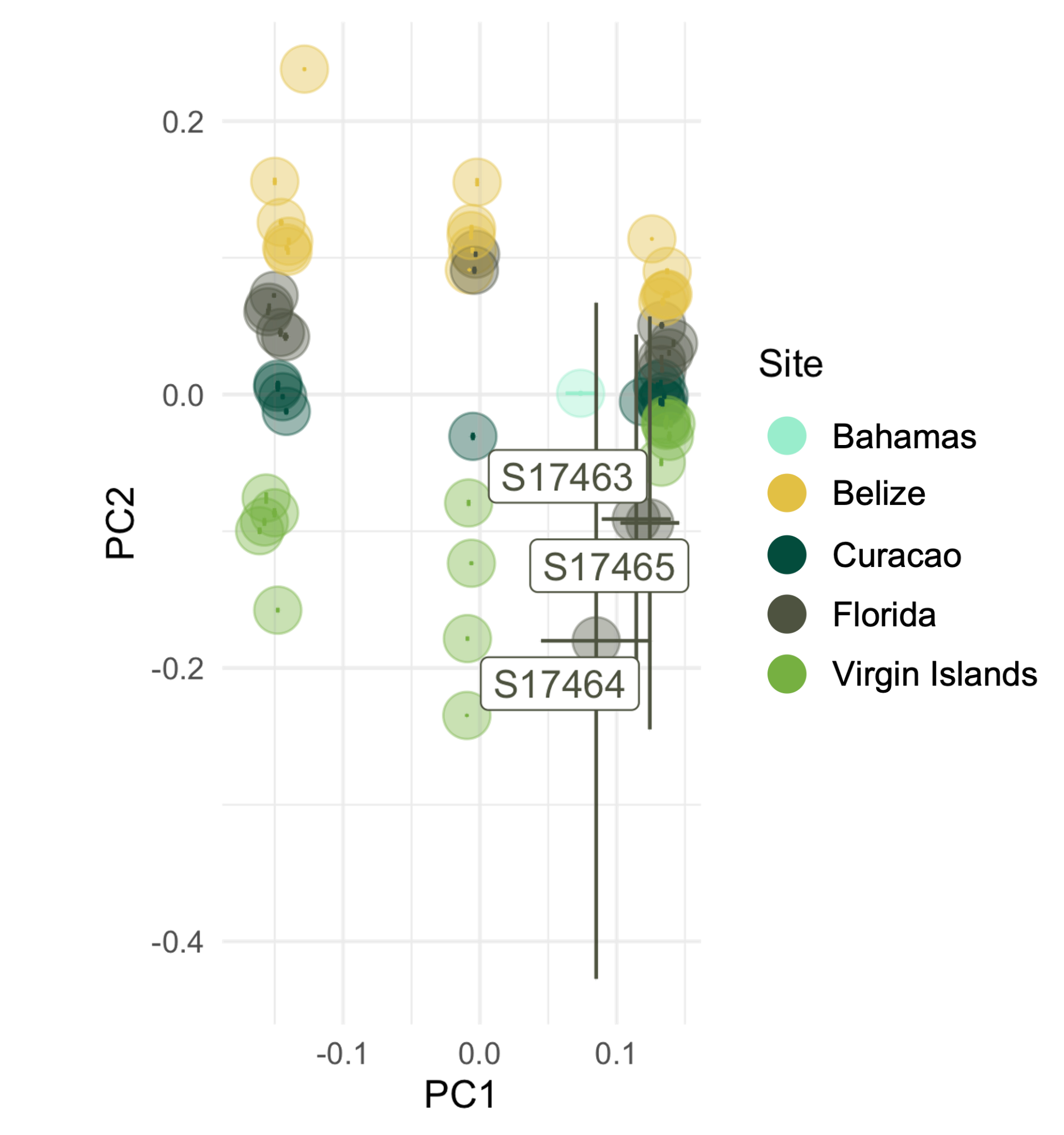


**Figure S3. PCA shows separation by site on PC2**

Projection-based PCA colored by sample origin. This recapitulates the structure found between sites in Kitchen *et al*. (2019)


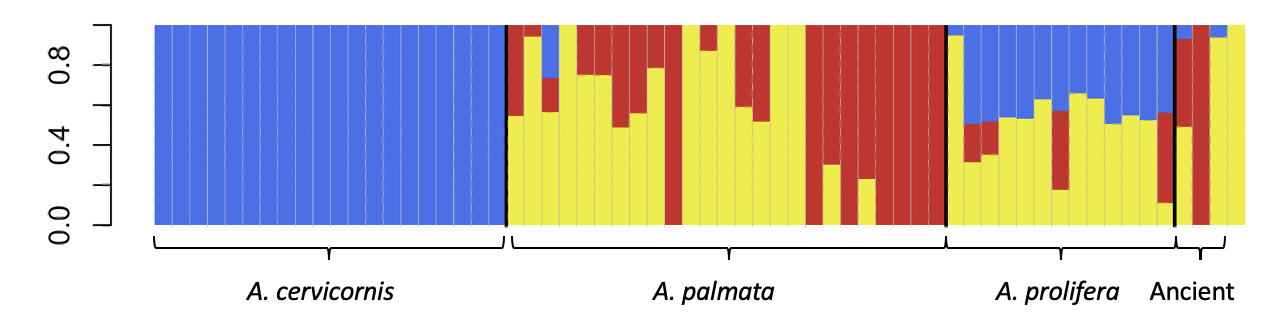


**Figure S4.** **Admixture plot with three clusters solidifies assignment of ancient samples to *A. palmata***

NGSAdmix plot for modern and ancient samples with K =3.


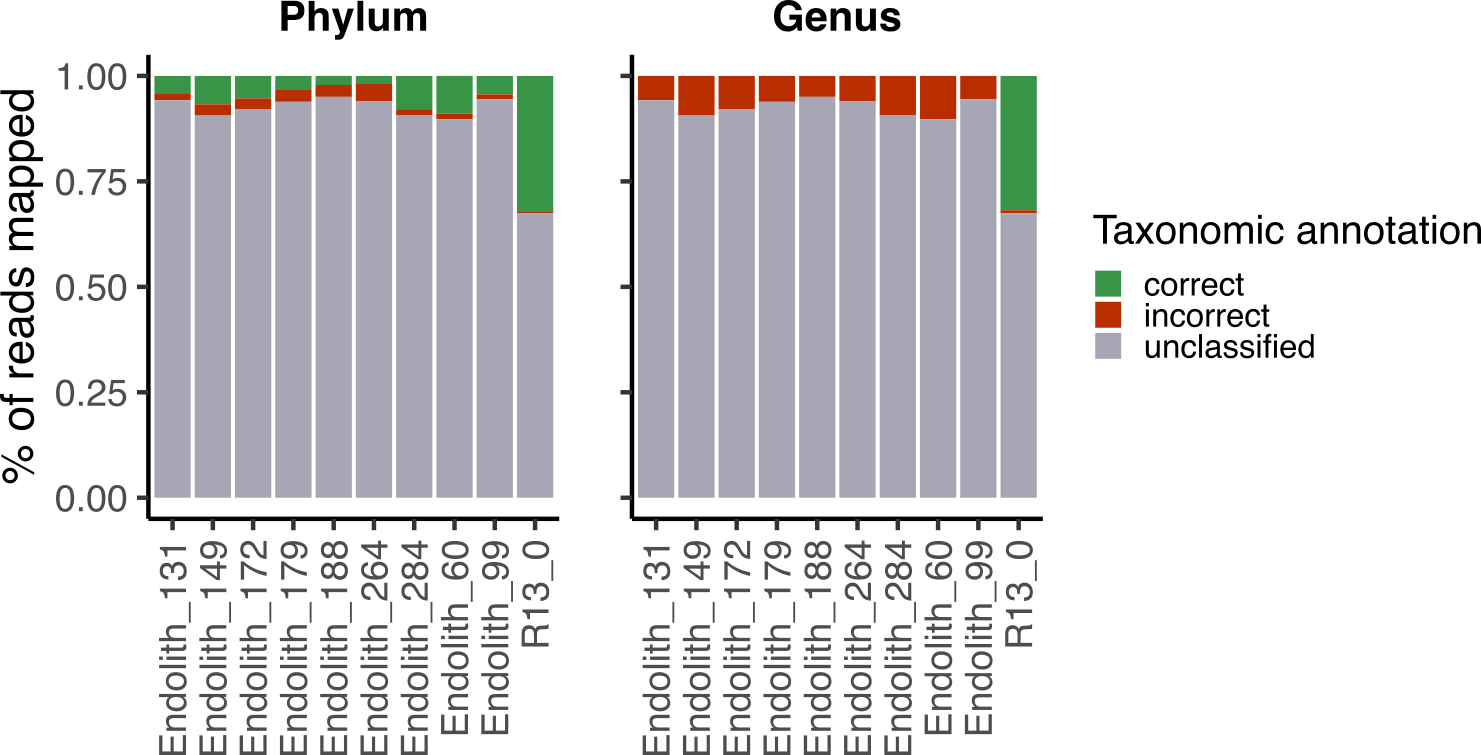


**Figure S5. Taxonomic validation of reads mapped to bacterial genomes.** Reads that mapped coral-associated bacterial genomes were taxonomically annotated using KAIJU. Taxonomic annotations were considered correct when the taxonomy assigned by KAIJU coincided with the genome taxonomy. Reads were unclassified if either did not map to any reference on the NCBI nr_euk database or ambiguously mapped to more than one genome of different taxa. The taxonomic level reported corresponded to the level at which the proportion of correct affiliations was higher than the incorrect ones.
